# PALS: peer support for community dwelling older people with chronic low back pain: a feasibility and acceptability study

**DOI:** 10.1016/j.physio.2019.01.015

**Published:** 2020-03

**Authors:** Kay Cooper, Patricia Schofield, Blair H. Smith, Susan Klein

**Affiliations:** a School of Health Sciences, Robert Gordon University, Aberdeen, UK; b Faculty of Health, Social Care & Education, Anglia Ruskin University, Chelmsford, UK; c Division of Population Health Science, University of Dundee, Dundee, UK; d Faculty of Health & Social Care, Robert Gordon University, Aberdeen, UK

**Keywords:** Peer support, Low back pain, Chronic pain, Self care, Self management, Adults

## Abstract

**Objectives:**

(i) Examine the feasibility and acceptability of a peer support intervention (PALS) to facilitate self-management in community dwelling older adults with Chronic Low Back Pain (CLBP), and (ii) examine the feasibility of study methods in order to inform the design of a future randomised controlled trial.

**Design:**

Mixed methods feasibility and acceptability study.

**Setting:**

Community.

**Participants:**

18 older adults (aged 65 to 79) with CLBP and 6 peer support volunteers (PSVs) aged 34 to 65.

**Intervention:**

Six sessions of 1 to 3 hours duration, approximately 2 weeks apart, delivered in mutually convenient public places, or by telephone. Each session had a suggested topic and each participant and PSV had a PALS manual detailing aims and target outcomes for each session.

**Outcome measures:**

Recruitment, retention, integrity, acceptability and feasibility of the PALS intervention, feasibility of study processes, appropriateness and usefulness of outcome measures.

**Results:**

We recruited to target and retained 2/3 of participants. PALS was delivered as intended and acceptable to people with CLBP and PSVs. Most participants were satisfied with PALS and would recommend it to someone else with CLBP. Study processes worked well, but recruitment procedures need to be refined. Outcome measures were returned and were mostly complete, but further work on the most appropriate measures is required.

**Conclusions:**

PALS was feasible to deliver and acceptable to the older people and PSVs who took part in this study. We identified amendments to PALS and the study processes that, once implemented, will allow the effectiveness of PALS to be tested in a large-scale study.

## Introduction

Low back pain causes more disability globally than any other condition, with prevalence and burden increasing with older age [1]. Chronic low back pain (CLBP: low back pain lasting beyond 12-weeks’ duration) is a common cause of disability in older adults [2], and the healthcare costs associated with CLBP are significant [3]. It is therefore important to develop effective methods of managing CLBP in older adults.

Many older adults with CLBP are managed by physiotherapists with evidence-based individually-tailored treatment aimed at facilitating self-management [4]. Self-management involves patients actively participating and taking responsibility for their condition to optimise function [5], but it can be difficult to achieve, with several reported barriers [6], [7]. Interest in methods of facilitating self-management has increased, with a growing evidence-base for peer support [8], [9].

Dennis defined peer support as “…the giving of assistance and encouragement by an individual considered equal” [10]. Peer support has been applied effectively in several chronic conditions and settings [e.g. 8,9,11,12]. However, a systematic review of peer support for chronic non-cancer pain found no evidence of peer support being tested in older adults with CLBP. It concluded that peer support may be more effective than usual care but highlighted the need for further high-quality research [13].

Peer support interventions vary according to the population and setting they are designed for, but are generally delivered by people who successfully manage the same health condition, and have received training in peer support [8], [9], [14], [17]. They can be delivered in group [14] or one-to-one formats [9], and by face-to-face [9], [15], telephone [8], [16] or internet-based [17] methods.

Contacts are usually 1 to 2 weeks [9], [15] and durations vary from a few months to years [9], [16], [18]. They aim to provide support to someone with a chronic health condition to facilitate self-management and coping strategies.

Peer support is therefore a potential method of facilitating self-management in older adults with CLBP; one that might provide a cost-effective solution for a subgroup of people with a common, costly and disabling condition. We developed and tested a peer support intervention (PALS) to facilitate self-management of low back pain in older adults following discharge from physiotherapy.

The aims of this study were to examine: (i) feasibility and acceptability of PALS to facilitate self-management and enhance health and wellbeing in community dwelling older adults with CLBP, and (ii) feasibility of study methods to inform the design of a future randomised controlled trial (RCT) of PALS. Ethical approval was granted by the North of Scotland Research Ethics Committee (13/NS/0094).

## Methods

### Design

**Fig. 1:**
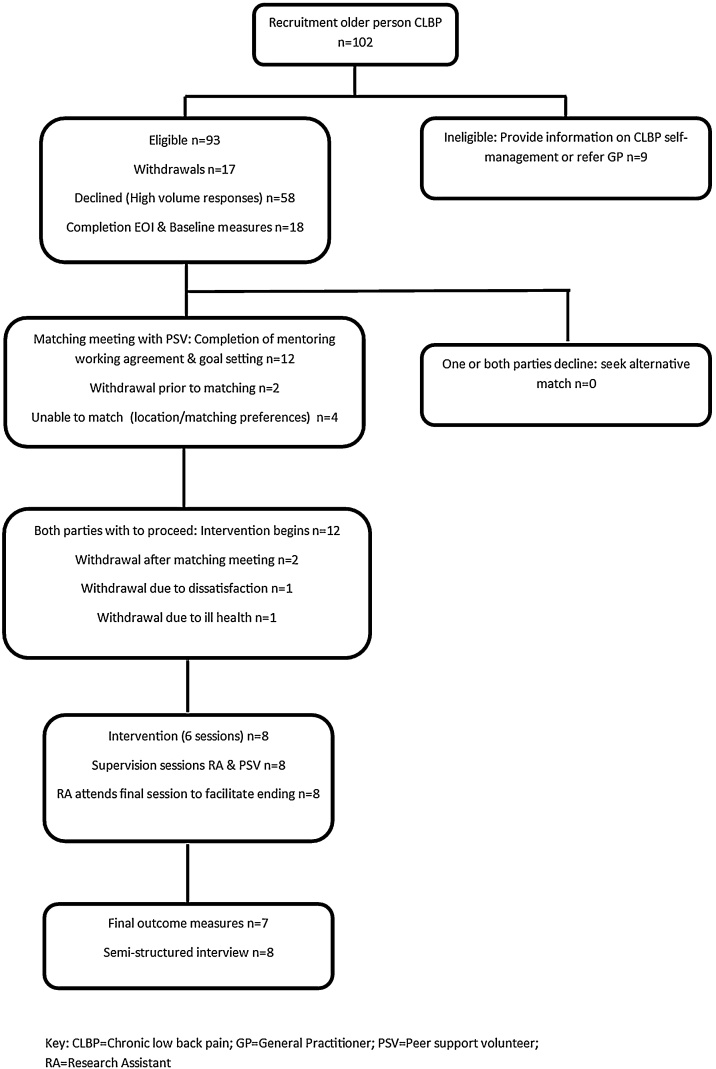
PALS study flowchart. CLBP = chronic low back pain; GP = general practitioner; RA = research assistant

We used sequential explanatory mixed methods to test a range of feasibility and acceptability measures. Fig. 1 outlines participant flow and study processes.

### Participants and recruitment

#### Older adults with CLBP

Sample size calculation was inappropriate for this feasibility study; instead, we based our target sample on previous research and time available for the study and aimed to recruit 10 to 15 participants on discharge from physiotherapy. Due to poor recruitment rates we also recruitedEligibility criteria were: (i) aged 65 years or older; (ii) received physiotherapy for CLBP (back pain of 12+ weeks’ duration); (iii) self-managing (not receiving treatments or interventions from healthcare professionals other than medication); and (iv) interested in receiving peer support. Exclusion criteria included ‘red flags’ indicative of serious spinal pathology and being unable to commit to the intervention. Initial telephone screening was followed by a second screening (telephone or face-to-face) where participants were asked: (i) what are your thoughts about PALS? (ii) what support do you hope a peer support volunteer (PSV) could provide? This information on CLBP self-management or to their general practitioner.

#### Peer support volunteers

Peer support volunteers were recruited from: (i) Previous study participants [19]; (ii) visits to local organisations (iii) press release. Inclusion criteria were: (i) aged 18+; (ii) have CLBP or experience of supporting someone with CLBP; (iii) live within 40-miles of the study centre; (iv) willing to commit to the PSV training and to support at least one older adult with CLBP. Full details of the PSV training are reported elsewhere [20]. Six PSVs who successfully undertook the training (1 male, 5 female) took part in this study

During a meeting with the study research assistant (RA) and after providing written, informed consent, each participant completed an expression of interest form. The RA identified possible matches, contacted each person separately, and arranged a matching meeting between the PSV, person with CLBP and RA. A mentoring working agreement was completed at this meeting, which included goal-setting for the older person with CLBP; a 7-day cooling-off period followed, during which either party could decline the match.

### PALS intervention

PALS was informed by: (i) a systematic review on peer support for chronic non-cancer pain [13]; (ii) a wider review of the literature; (iii) consultation with individuals and organisations experienced in peer support for chronic health conditions, (iv) a qualitative study exploring older adults with CLBP and physiotherapists’ perceptions of peer support [19].

**Fig. 2:**
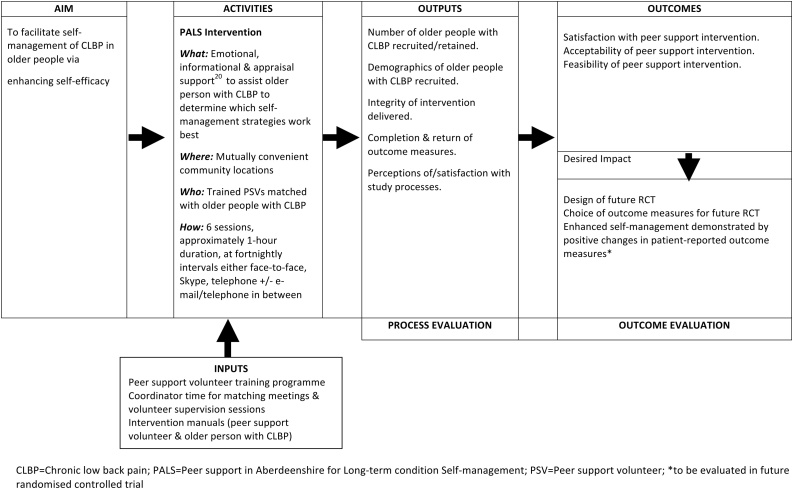
PALS peer support intervention logic model. CLBP = Chronic low back pain; PALS = peer support in aberdeenshire for long-term condition self-management; PSV = peer support volunteer; *to be evaluated in future randomised controlled trial.

Fig. 2 details the logic model for the study. Full details of PALS is available in supplementary file 1. PALS was underpinned by empowerment theory [15] and aimed to facilitate CLBP self-management by enhancing self-efficacy [14], [16], [18] and maintaining/increasing physical activity [4]. It consisted of 6 individually-tailored sessions delivered at fortnightly intervals in mutually convenient public places, or by telephone/Skype. All participants received a manual (available from the author on request) detailing aims, target outcomes, suggested preparation for sessions, and self-management resources [e.g. [21], [22], [23], [24]]. Participants completed an activity log between sessions and PSVs received a short telephone supervision following each session to monitor intervention fidelity; in order to preserve the peer support nature of PALS, CLBP participants did not receive this support. The RA attended the end of the final session to facilitate ending the peer support process; we deemed this appropriate, as a peer support coordinator would likely fulfil this role in future.

### Measures

By monitoring study processes and analysing telephone supervision, activity logs, post intervention semi-structured interviews (Supplementary file 2), and satisfaction questionnaires (Supplementary file 3) we explored feasibility and acceptability of PALS and feasibility of study processes.

The following standardised outcome measures were selected, based on their use in previous studies of peer support and/or self-management of CLBP and the constructs of interest: Roland Morris Low Back Pain Disability Questionnaire (RDQ [25]) numerical rating scale [26] for pain intensity; EuroQol EQ-5D [27] for quality of life; Pain Self-Efficacy Questionnaire (PSEQ [28]) Warwick Edinburgh Mental Wellbeing Scale (WEMWBS [29]); physical activity stage of change (SOC [30]). Measures were completed pre-intervention during the face-to-face meeting with the RA. Post intervention measures were given to participants at the end of the semi-structured interview along with a freepost envelope for return. Two participants had difficulty with writing; the RA completed these measures as a structured interview. Post intervention measures also included Global Impression of Change [31].

### Data processing and analysis

Descriptive statistics were calculated for feasibility measures. Interviews were transcribed and data mapped onto Framework matrices, arranged according to interview topics. Due to the structured nature of the interviews, data was not coded prior to mapping [32]. Data analysis followed the Framework approach [33] and was conducted by two researchers.

## Results

***Recruitment and retention*** are summarised in Fig. 1. No participants were recruited on discharge from physiotherapy; 2 press releases resulted in 93 eligible participants 18 were recruited (8 male, 10 female) and 58 declined, due to reaching target recruitment.

Of the 18 participants recruited, all were retired, their ages ranged from 65 to 89 years (mean 75), and all had a long history (2 to 50 years) of constant or recurrent back pain. had co-existing health conditions such as Parkinson’s disease; coronary heart disease; fibromyalgia; depression and/or anxiety. Recruitment of PSVs is reported elsewhere [20]. The six included here (1 male; 5 female) were aged 34 to 65 years (mean 54). Three were retired, and three were in full or part-time employment. All had at least 10 years’ history of CLBP.

Of the 18 participants recruited, 12 were matched with PSVs and 8 completed the interventionTwo withdrew after the matching meeting, stating that the information in the manual was sufficient for them to self-manage their CLBP. One match proved unsuccessful because, after agreeing to communicate by e-mail and telephone this proved unsatisfactory for the person with CLBP.

### Integrity & feasibility of PALS

All PSVs delivered 6 sessions of 1 to 3 hours’ duration approximately 2-weeks apart. Most partnerships met face-to-face in mutually convenient community locations. Two partnerships substituted one or two meetings with telephone calls.

Some partnerships worked through the PALS manual, covering a different topic at each meeting and reflecting on the resources each had consulted. Others took a less formal approach discussing what was of most concern that week. Some PSVs required prompting by the RA to revisit the goals set at the matching meeting.

Discussion centred on each other’s experiences of CLBP self-management and their thoughts on the information in the manual. PSVs provided encouragement to begin/continue with strategies related to goals set at the matching meeting, and provided a *“sounding board”* for participants to talk about their CLBP, and in several cases other problems (e.g. family). Some participants tried new self-management strategies, with encouragement from PSVs, such as exercises, walking, and water-based exercise. Three partnerships incorporated physical activity into their meetings.

Six participants completed activity logs; one participant had poor sight and difficulty writing, therefore declined to complete it one “*just kept forgetting”*, and one discontinued as she felt it was repetitive. The remaining logs detailed exercises, physical activities and self-management strategies utilised throughout the week as well as participants’ thoughts on their usefulness, medication changes, and healthcare visits (very few). The RA provided reassurance to PSVs during the supervision sessions, who were at times unsure whether they were doing things “right”. No problems were reported during the study.

### Acceptability of PALS

Supplementary file 3 details satisfaction questionnaire results, returned by 7 participants. Most participants were satisfied with most aspects of PALS. Five would recommend PALS to someone else with CLBP (one missing item).

### Feasibility of study processes

Fig. 1 details completion of baseline and follow-up measures. One telephone reminder was required for follow-up measures, and 1 participant failed to return them despite a postal and voice-mail reminder.

Of the 12 participants who completed baseline measures, 3 did not complete the numerical rating scale for pain and 2 had missing items on the WEMWBS. Of the 7 who completed follow-up measures, 1 had a missing item on the EQ-5D and 1 on the satisfaction questionnaire.

### Appropriateness and usefulness of outcome measures

Table 1 presents outcomes for each participant. Because of the small sample size it is inappropriate to make inferences from these findings. Individual EQ-5D and WEMWBS scales are not presented in Table 1; in contrast to the NRS for pain, no participants scored worse at follow-up for EQ-5D pain/discomfort scale. Only one participant scored worse at follow-up for the EQ-5D self-care scale. Similarly, for the WEMWBS score, only one participant scored worse for each of the following subscales: “I’ve been feeling interested in other people”, “I’ve been feeling close to other people” and “I’ve been feeling cheerful”.

**Table 1:** Baseline (Pre) and follow-up (post) outcome measures.

Participant	RDQ Pre (post)	NRS Pre (post)	EQ-5D HS Pre (post)	PSE Pre (post)	WEMWBS Pre (post)	PASOC Pre (post)	GIC post
**1**	**10 (5)**	2 (2)	70 (50)	**55 (58)**	45 (44)	5 (3)	**Better**
**2**	10 (11)	5 (7)	44 (35)	36 (31)	**44 (45)**	5 (5)	A little worse
**3**	10 (10)	**5 (3)**	80 (80)	**47 (51)**	55 (55)	3 (3)	**Better**
**4**	3 (5)	3 (6)	80 (60)	48 (45)	54 (51)	4 (3)	A little worse
**5**	**5 (3)**	5 (5)	**70 (75)**	**39 (45)**	**52 (60)**	**5 (5)**	**A little better**
**6**	14 (20)	5 (6.5)	50 (50)	**38 (39)**	51 (44)	**2 (3)**	No change
**7**	9 (13)	**3 (2)**	60 (50)	42 (33)	42 (38)	3 (3)	**A little better**

RDQ = Roland Morris Low Back Pain Disability Scale (0 to 24, high score indicates higher disability); NRS = numerical rating scale for pain (0 to 10, high score indicates higher pain); EQ-5D HS = EQ-5D health scale (0 to 100%, higher score indicates better health); PSEQ = Pain self-efficacy questionnaire (0 to 60, higher score indicates greater confidence in managing despite pain); WEMWBS = Warwick-Edinburgh Mental Wellbeing Scale (7 to 70, higher scores indicate better wellbeing); PASOC = Physical Activity Stage of Change (1 to 5, higher scores indicate greater physical activity); GIC = Global Impression of Change. Bold indicates improvement.

### Semi-structured interviews

The 4 PSVs who delivered the intervention in full (1 male, 3 female) and 8 participants who received it (3 male, 5 female) took part in interviews. Findings related to 4 key topics, 3 of which are discussed below, with representative quotes for each topic presented in Table 2. The fourth topic related to PSVs’ perceptions of the training [20].

**Table 2:** Representative quotes from one-to-one interviews.

	People with CLBP	Peer support volunteers
**Topic 1: expectations**	*“Hoping I could get more encouragement of what I should be doing”* [P52, Male]	*“Gone part time and I was looking for things to fill up my day. Also because I had experienced a pain – knees and hips, maybe I could learn from other people or maybe I could help them. Thought it was something good to be involved in”* [PSV47, Female]
	*“Thought meeting someone with similar problems would help accept how you were yourself and maybe offer suggestions”* [P70, Female]	

**Topic 2: the intervention**	***Matching***	
	*“[volunteer] was a lot younger than me but it didn’t matter, we both had active lives, we had a lot to relate to”* [P56, Male]	*“You don’t have to have a lot of other things in common if you both have back pain, both have an understanding”* [PSV66, Male]
	***Delivery***	
	*“Both [face-to-face & telephone] were good…just as easy over the phone…but it’s vital to see a face, you couldn’t do them all by phone”* [P52, Male]	*“You could do it on phone but I need to see a person. I like meeting up. If the patient's at their home and on the phone they can't get things off their chest.”* [PSV42, Female]
	*“A week apart would be too fast, no time to put anything into practice…I also think an hour is long enough, it’s long enough for most people’s concentration spans.”* [P70, Female]	*“Body language is important. The physical thing too of getting out, it's an activity that gets you out and moving. So it could be negative just sitting at home doing Skype and e-mail, it's supposed to be active [back pain self-management].”* [PSV66, Male]
	*“[six sessions were] enough, felt it was time to finish, I was accomplishing what I could get out of it”* [P52, Male]	
	***What I got out of it***	
	*“It forced me to have an action plan…I’m doing exercises now and they are really helping”* [P48, Male]	*“Think I got as much out of it as the patients have. I learned a lot about pain and different people's pain thresholds, ways of managing. Think I’m more tolerant of back pain as a result of the study”.* [PSV40,Female]
	*“It gave me a wee push…although I am much the same I think about it more [back pain and self-management strategies]* [P57, Female]	*“ I learned some new things − pacing was good”*. [PSV47, Female]
	*‘Just somebody listening to you, getting a few things off your chest’* [P67, Female]	
	*“Don’t underestimate the importance of psychological support…sympathetic, encouraging, that was the biggest benefit…encouraged me to keep going with what I do already…encouraged me to not get too overwhelmed” [*P70, Female]	
	***The intervention manual***	
	*“Useful − I made a point of looking at it before & after session. Without the plan of action we would have wandered a lot”* [P48, Male]	*“The best thing I found was the manual it gave criteria to work to. If the patient went off on a tangent I could bring it back to focus using the manual and topic for that session…but the content could be halved”* [PSV66, Male]
	*“ A lot of the paperwork was repetitive…it’s not necessary to repeat. The matching meeting at the start explained it well…you need a note of how you felt it went each time, a certain amount of recording but not repetitive questions…we did look at some of the leaflets together…the Mental Health booklet was interesting…and not filled with little drawings, much more factual.* [P70, Female]	*“The resources were good but quite laborious for the patient, especially if they didn't like using computers…Pacing was good and dealing with pain. I used the bit ‘what are you hoping to achieve’ tried to go by that. We did end up speaking about other things but used it as a guide.”* [PSV47, Female]
	*‘Writing the activity diary was useful, it showed how I did too much, I could see where I should be relaxing more.’* [P67, Female]	
**Topic 3: study processes**	***Support***	
	*“Good support from the study team.”* [P68, Female]	*“The support was very helpful. Phone-calls after the sessions were helpful.”* [PSV66, Male]

### Topic 1: Expectations

Participants had no previous experience of peer support and no expectations of what the study might involve. Motivation for PSVs to take part came from wanting to try new things, meet other people with CLBP, and thinking they might benefit as well as helping others. Motivation for CLBP participants was related to the hope of benefit to their symptoms or gaining a better understanding of CLBP.

Two participants thought there might be health professional involvement, suggesting that recruitment materials and study information should be reviewed before using in a future RCT. One participant said: *“It maybe was clear enough, it was maybe just me, hoping that there was maybe something else”* [P69: Female]. The screening questions for recruitment may have been unsuccessful for this participant, who may not have been fully ready to engage with a self-management approach.

All PSVs said they benefited from taking part and learned new things, particularly in relation to pacing of activities.

### Topic 2: the intervention

i Matching: Participants were generally positive about their matches. With the exception of two female participants who requested a female PSV, they said that gender and age were not important. It was more important to “get on” and have something in common (CLBP).

Two participants matched with the same PSV commented that she was at times too general in her approach, although they both liked the volunteer personally. This suggests that there is a balance to be found between being friendly and having some structure to the meetings, and that these participants were looking for more than just a befriending service, supporting the structured nature of PALS. However, the other participant matched with this PSV was happy with the match. Matching is therefore an inexact process and it may not be possible to find ideal PSVs for each participant; it may be worth exploring during the training how PSVs might do things differently with the different partners they become matched with.

ii Delivery: All participants, including those who had one or more telephone meetings, felt that a face-to-face element was essential. Participants were generally satisfied with the timing and dosage of PALS.
iii What I got out of it: One CLBP participant felt PALS did not help her at all; this participant hoped there would be healthcare professional involvement (discussed above). The remaining participants reported benefit, although not always in the way they had anticipated. This tended to be the case for those who expected practical support but found they benefited more from emotional support. These comments suggest that, although participants may not have had large benefits in terms of objective outcomes, they perceived a benefit from the peer support.
iv PALS Manual: Participants spoke variably of the manual and resources, with some liking the information provided, some using the manual as a step-by-step guide, and some not using it at all. It was felt that the manual could be reduced in volume, that repetitive elements could be removed, and that one or two resources were sufficient.

### Topic 3: study processes

Participants reported no issues with study processes (recruitment, communication with study team, paperwork, outcome measurement). Three participants would have taken part had it been an RCT, as they had been involved in research before and understood the need for randomisation; the other four were unsure (one non-responder). Testing peer support using RCT methodology will require careful thought when preparing study recruitment materials and procedures, such as considering a patient preference design. interviewees would recommend PALS to other people with CLBP, several thought that peer support would be useful for other health conditions, and all PSVs would like to be involved in delivering the intervention again.

## Discussion

PALS was feasible and acceptable to participants and study processes were feasible. However, prior to scaling up to a large-scale RCT to test the effectiveness of PALS, it will benefit from some modifications. We recruited to target and retained 2/3 of participants: slightly less than previous research on peer support for veterans with chronic musculoskeletal pain [8], nonetheless, we considered this acceptable given the genuine reasons for withdrawal. These reasons suggested that inclusion criteria should be refined prior to conducting a large-scale study and that withdrawals were likely due to general population recruitment rather than the intended strategy of recruiting from physiotherapy departments.

The lack of recruitment from physiotherapy departments can be attributed to study commencement coinciding with major service redesign and staff shortages in out-patient departments. Whilst physiotherapists were supportive of the study they found it difficult to find time to recruit participants. Careful thought is required in the design of an RCT, particularly dedicating funded staff time for participant recruitment. Recruiting from the general population was successful and arguably appropriate as the prevalence of CLBP [2] suggests there are many people living with CLBP in the community but not currently accessing services who might benefit from peer support. Nonetheless, these self-selected participants may differ from older people recruited from physiotherapy departments.

The matching process worked well and was not dependent on age or gender-matching of participants. There is little discussion of the matching process in the peer support literature. We found it to be a somewhat inexact process, largely dependent on the judgement of the RA. Although some participants expressed preferences in terms of age, gender and interests, these preferences could not always be accommodated.

PALS was delivered as intended and the dosage was acceptable to our participants. Matthias *et al.* [8] recommended 8 sessions in their 4-month peer support intervention but found the median number of sessions delivered to be 6, the same as our study. Clearly the optimum dosage needs further evaluation, and flexibility of dosage might better support person-centred care [34].

Our participants agreed that at least some meetings had to be face-to-face. Previous research has however successfully delivered peer support exclusively via telephone [16]. This finding can likely be attributed to our small sample, and it is our intention to further test PALS delivered face-to-face, by telephone/Skype and combined methods, allowing participants with access or transport barriers to benefit from the intervention.

The manuals were of benefit but require some refinement prior to further study, particularly information and web-based resources. Interestingly, we offered the resources (and outcome measures) in electronic format, which we believed to be in keeping with the increasing interest in digital health interventions [35]. No participants chose the electronic versions of the manual or outcome measure completion, suggesting that there is still a need for paper-based materials in health interventions and research.

The study processes were feasible and acceptable to participants. Outcome measures were completed in full by most participants. Our small sample size prevented the drawing of specific inferences from the results, but they indicate that EQ-5D and WEMWBS subscales, self-efficacy and PGIC might be meaningful outcomes. Matthias *et al.* [8] demonstrated positive effects of peer support on Patient Activation [36] and Pain Centrality [37], both of which should be considered for a future RCT. The interview finding of social and emotional support being a key, sometimes unexpected, positive feature of PALS supports previous research [8] and Dennis’s definition of peer support [10]. The challenge is perhaps in adequately capturing this aspect in outcome measurement. Several participants felt they benefited from PALS, attributing the benefit to the social and emotional aspects of peer support, but their outcome measures did not necessarily demonstrate improvements. This is not uncommon in the field of peer support [38] [e.g. 38], and supports the need for further development of outcome measures for an RCT. It may however be a result of broad inclusion criteria resulting in confounding from the presence of co-morbidities. The future RCT should carefully consider inclusion criteria in light of this, but also in light of the increasing prevalence of co- and multi morbidity in the ageing population.

As with previous research [39], our PSVs appeared to benefit from involvement in the study; this finding can be used to facilitate recruitment of further PSVs to deliver interventions of this kind.

This study has several limitations. The sample size was small and drawn from one geographical location of the UK. Whether PALS would be suitable for use elsewhere and with a more diverse sample of people with CLBP and PSVs requires further study. We were unable to interview two PSVs who did not deliver the full intervention, and the two people with CLBP who withdrew, therefore it is possible that alternative viewpoints have not been captured in the interviews.

## Conclusion

We have demonstrated the feasibility and acceptability of a peer support intervention for a sample of older people with CLBP. We have identified amendments to be made to the intervention and study processes before a full evaluation can be conducted, namely: (i) addressing recruitment from physiotherapy departments; (ii) reviewing screening processes and inclusion/exclusion criteria; (iii) refining PALS manuals; and (iv) reviewing outcome measures. In keeping with Medical Research Council (MRC) guidance for the development of complex interventions [40], this will allow the effectiveness of PALS to be tested in a large-scale study.

**Key messages**

• We have demonstrated that peer support for older people with chronic low back pain is feasible to deliver and acceptable to older people and peer support volunteers.
• We have identified aspects of the peer support intervention and study processes that should be improved prior to further testing in a large-scale study.

## Acknowledgements

The authors would like to thank Mary Llinos Jehu and Susan Massie who assisted with data collection, processing and analysis.  *Ethical approval*: North of Scotland Research Ethics Committee (No: 13/NS/0094).  *Funding*: This work was supported by The Dunhill Medical Trust [grant number R300/0513].  *Conflict of interest*: None declared.
