# Supplementary material for: PALS: peer support for community dwelling older people with chronic low back pain: a feasibility and acceptability study
Source: Physiotherapy. 2020 Mar;106:154–62. doi: 10.1016/j.physio.2019.01.015 (PMC7029274; doi:10.1016/j.physio.2019.01.015)
Supplement: Supplementary file 1 [file mmc1.pdf]

Supplementary File 1: Content of PALS Intervention

Session	Topic	Description
1	Living with persistent low back pain	Exploring functional, social & emotional aspects of living with CLBP
2	Exercise & Physical Activity	Exploring the role of physical activity for CLBP and opportunities for being physically active
3	Adhering to self-management strategies	Exploring self-management strategies for CLBP
4	Goal setting & action –planning	Exploring how to put things into action
5	Problem-solving & flare-ups	Exploring problem-solving and what to do in the event of a flare-up
6	Review and ending the peer support relationship	Preparing for longer-term self-management
