# Supplementary material for: PALS: peer support for community dwelling older people with chronic low back pain: a feasibility and acceptability study
Source: Physiotherapy. 2020 Mar;106:154–62. doi: 10.1016/j.physio.2019.01.015 (PMC7029274; doi:10.1016/j.physio.2019.01.015)
Supplement: Supplementary file 2 [file mmc2.pdf]

Supplementary file 2: Interview Topic Guide

Main Topic	Example prompts used
**People with CLBP**	
Expectations	How they found out about study<br>Had they heard of peer support previously<br>What did they expect to happen/what did happen
Study completion	What did they think of matching process<br>How many sessions were attended
The intervention	Mode of delivery<br>What they did/discussed during the sessions<br>What they thought of the sessions<br>What they thought of the manual<br>What they thought of the ending process
Influence on low back pain self-management	Was the intervention helpful/not helpful for them<br>Would they recommend to others with low back pain<br>What could be done differently<br>Any ideas on when & how to introduce peer support to a patient journey
Study Processes	What did they think of the forms (data collection forms/outcome measures)<br>What did they think of the support from the research team
Future Study	If this had been an RCT would they have taken part
Anything else	Anything else they would like to reflect on
**Peer Support Volunteers**	
Expectations	How they found out about study<br>Had they heard of peer support previously<br>What did they expect to happen/what did happen
Intervention	How many people they supported<br>What did they think of matching process<br>What did they think of resources<br>How did the conduct the intervention<br>What did they do/discuss during sessions<br>What did they think was effective/not effective<br>What did they think of the manual<br>Has it affected their self-management
Study processes	What did they think of support provided by research team<br>What did they think of paperwork
Anything else	Anything else they would like to reflect on
