# Supplementary material for: PALS: peer support for community dwelling older people with chronic low back pain: a feasibility and acceptability study
Source: Physiotherapy. 2020 Mar;106:154–62. doi: 10.1016/j.physio.2019.01.015 (PMC7029274; doi:10.1016/j.physio.2019.01.015)
Supplement: Supplementary file 3 [file mmc3.pdf]

Supplementary File 3: Results of Satisfaction Questionnaire (n=8)

Satisfaction with:	Satisfied	Dissatisfied	Neutral
Information I was provided with before the intervention started	5	0	2
Information I was provided with during the course of the intervention	6	0	1
Matching with the peer support volunteer	6	0	1
Communication with the peer support volunteer	5	0	2
Communication with the project co-ordinator	5	1	1
Support I received from the peer support volunteer	5	0	2
Support I received from the project co-ordinator	4	1	2
Ending the peer support relationship	6	0	1

Note: All items presented as 5-point Likert scales; "Very satisfied" and "satisfied" are combined here as "satisfied";"very dissatisfied" and "dissatisfied" are combined here as "dissatisfied"
