# Supplementary material for: PALS: peer support for community dwelling older people with chronic low back pain: a feasibility and acceptability study
Source: Physiotherapy. 2020 Mar;106:154–62. doi: 10.1016/j.physio.2019.01.015 (PMC7029274; doi:10.1016/j.physio.2019.01.015)
Supplement: Supplementary file 4 [file mmc4.pdf]

Would you like some support to help you manage your back pain?

Health researchers from Robert Gordon University's School of Health Sciences have developed a peer-support intervention for older people with chronic low back pain, and are looking for some volunteers to help them test it out.

The team, led by Dr Kay Cooper, have developed the intervention and trained several peer-volunteers to deliver it, all of whom have experience of living with low back pain themselves.

To be eligible for the study you need to be 65 or over, living in or within 20 miles of Aberdeen City, and have low back pain lasting 3 months or longer for which you are not currently receiving any formal treatment or interventions. The intervention is however ideal for people who have recently received physiotherapy, as it is designed to be a stepping-stone between formal treatment and self-management.

Taking part in the research would involve being matched up with a trained peer support volunteer and receiving their support via six meetings or phone conversations, or a series of e-mail exchanges if you prefer, over a 2-3 month period.

The researchers would collect a range of measures during this period to find out whether the intervention is appropriate and feasible to deliver and receive, as well as how effective people think it is in helping them to self-manage their back pain.

If you would like to find out more, or might be interested in taking part in the research please contact Dr Kay Cooper on [telephone number & e-mail address supplied]
